# miR–122–5p Promotes Cowshed Particulate Matter2.5-Induced Apoptosis in NR8383 by Targeting *COL4A1*

**DOI:** 10.3390/toxics12060386

**Published:** 2024-05-25

**Authors:** Yize Sun, Ke Sun, Zhenhua Ma, Xiqing Zhang, Xiaohui Du, Yunna Jia, Yanbin Zhu, Muhammad Inam, Yunhang Gao, Wangdui Basang

**Affiliations:** 1 Department of Veterinary Medicine, College of Animal Science and Technology, Jilin Agricultural University, Changchun 130118, China; sunyize1228@163.com (Y.S.);; 2 Institute of Animal Husbandry and Veterinary Medicine, Tibet Academy of Agricultural and Animal Husbandry Science, Lhasa 850009, China; 3 Department of Zoology, Shaheed Benazir Bhutto University Sheringal, Dir Upper 18050, Pakistan

**Keywords:** PM_2.5_, microRNA, ECM, PI3K/AKT, apoptosis

## Abstract

It is well known that Particulate Matter2.5 (PM_2.5_) has a major adverse effect on the organism. However, the health hazards of livestock farm PM_2.5_ to humans and animals are not yet known, and the role of miRNAs in the cellular damage induced by livestock farm PM_2.5_ is also unclear. Therefore, our study used cowshed PM_2.5_ to stimulate rat alveolar macrophage NR8383 to construct an in vitro injury model to investigate the effect of miR–122–5p on PM_2.5_-induced apoptosis in the NR8383. The level of apoptosis was quantified by flow cytometry and Hoechst 33342/PI double staining. Furthermore, the potential target gene Collagen type IV alpha (*COL4A1*) of miR–122–5p was identified through the use of bioinformatics methods. The results demonstrated a decline in cell viability and an increase in apoptosis with rising PM_2.5_ concentrations and exposure durations. The transfection of miR–122–5p mimics resulted in an upregulation of the pro-apoptotic protein Bcl–xL/Bcl–2 and activation of cleaved caspase–3 while inhibiting the anti-apoptotic protein B–cell lymphoma–2. The experimental data indicate that miR–122–5p is involved in the apoptotic process by targeting *COL4A1*. Furthermore, the overexpression of *COL4A1* was observed to enhance the PM_2.5_-activated PI3K/AKT/NF–κB signaling pathway, which contributed to the inhibition of apoptosis. This finding offers a promising avenue for the development of therapeutic strategies aimed at mitigating cellular damage induced by PM_2.5_ exposure.

## 1. Introduction

Air pollution has emerged as a significant public health concern in recent years. PM_2.5_, a particulate matter with a diameter of 2.5 microns or less, is a prevalent air pollutant that poses irreversible harm to both humans and animals [1]. Extensive studies have established that long-term exposure to PM_2.5_ can lead to the development of asthma, cardiovascular diseases, and chronic obstructive pulmonary diseases, among others [2,3,4]. This pollutant is not only found in industrial and urban areas but also where animal husbandry is conducted. The breeding industry is very important all over the world, and the harm caused by cowsheds and breeding houses in environmental pollution has an impact on the whole world [5,6]. In recent years, the rise of intensive farms in China has prompted widespread concern over the presence of PM_2.5_ in these vicinities.

Other studies have indicated a close correlation between PM_2.5_ and respiratory illnesses [7]. Alveolar macrophages are an integral component of the immune system and are located along the sides of the lung cavity [8]. They constitute the first line of defense for clearing pathogens and pollutants from the lungs and coordinating the initiation and cessation of immune responses within the respiratory system. The continuous exposure to PM_2.5_ exacerbates damage to the alveolar macrophages, ultimately resulting in apoptosis. Some reports suggest that the extracellular matrix (ECM) around the macrophages provides structural support for cellular adhesion and the perception of physical stimuli [9]. Collagen, the most abundant protein in the human body, may be involved in cellular damage [10]. Here, we discuss the role of the ECM in the apoptosis of alveolar macrophages.

Collagen type IV alpha is the primary component of the extracellular matrix basement membrane. Collagen IV measures 400 nm in length and possesses three chains, which are composed of two α I (IV) chains and one α Ⅱ (IV) chain [11]. Recent studies have demonstrated a correlation between *COL4A1* and cardiovascular diseases, indicating that it may alleviate cell damage [12,13]. Furthermore, collagen type IV has been reported to impact the respiratory system and can be found in asthma and lung tumors [14,15]. To sum up, these findings suggest that *COL4A1* has significant implications for both cardiovascular and respiratory disorders. However, there is minimal research on the precise mechanism linking *COL4A1* and respiratory illnesses. Thus, it is crucial to conduct a thorough investigation of the regulatory process between *COL4A1* and alveolar macrophage apoptosis and identify the gene targets for future disease prevention and clinical treatment.

MicroRNA is one of the many known regulators of *COL4A1*. MicroRNAs (miRNAs) are short non-coding RNAs that modulate gene expression by interacting with mRNAs, resulting in mRNA decay or suppression of translation [16]. Typically, miRNAs are endogenously expressed at the cellular level, and they combine with mRNA for transcription and translation while regulating multiple physiological functions, such as cell development, proliferation, and apoptosis [17]. It was found that a variety of miRNAs are expressed to varying degrees under PM_2.5_ exposure [18]. Extensive data demonstrate the ability of miR–122–5p to regulate renal fibrosis in vivo and promote tumor and cancer cell proliferation [19,20]. However, knowledge of miR–122–5p’s impact on respiratory diseases is limited. As a result, we investigated the precise control mechanisms of miR–122–5p that influence apoptosis initiated by PM_2.5_ in cowsheds.

Several cell functions are typically affected by the phosphatidylinositol 3kinase (PI3K)/protein kinase B(AKT) signaling pathway, which is one of the classic pathways that govern apoptosis in organisms [21,22,23]. It is usually mediated by the phosphorylation of serine or threonine in the downstream stage. The PI3K/AKT signaling pathway is associated with a large number of human diseases, such as cancer (tumors), lung injury, and so on [24]. Other studies have shown that lung injuries caused by other diseases can be alleviated by interfering with the PI3K/AKT/NF–κΒ signaling pathway [25]. Therefore, we have put forward the following hypothesis: apoptosis induced by cowshed PM_2.5_ is also associated with the PI3K/AKT/ NF–κΒ signaling pathway.

Cowshed PM_2.5_ had the first effect on apoptosis in our study. The *COL4A1* and PI3K/AKT/NF–κΒ pathways were found to be the mechanisms by which miR–122–5p could interfere with cells during apoptosis in this process. Our research provides a theoretical framework for studying the influence of the agricultural environment on cell functions and lays a background for further research in this area.

## 2. Materials and Methods

### 2.1. PM_2.5_ Collection

We used cowshed PM_2.5_ collected by our research group in the early stage [26]. The PM_2.5_ particles were crushed using ultrasound and fully mixed with normal saline to prepare a suspension. The resultant samples were stored at −20°C for further use.

### 2.2. Cell Culture and Treatments

Rat alveolar macrophages (NR8383) and human renal epithelial cells (HEK-293T) were sourced from the Shanghai Cell Bank, Shanghai, China. The cultures were maintained in a humidified incubator with a 5% CO_2_ atmosphere at a constant temperature of 37 °C. The growth medium consisted of Dulbecco’s modified Eagle medium (DMEM; Gibco, Gaithersburg, MD, USA), supplemented with 10% fetal bovine serum (FBS; Gibco) and 1% penicillin–streptomycin (Beyotime Biotechnology, Shanghai, China).

The experimental design included two distinct groups:

1. The cells were exposed to escalating concentrations of cowshed PM_2.5_, ranging from 0 μg/mL to 300 μg/mL in increments of 60 μg/mL.
2. A fixed concentration of PM_2.5_ (180 μg/mL) was used to treat cells for varying durations: 0, 12, 24, and 48 h.

### 2.3. Cell Viability (CCK-8)

Cell viability was assessed using the CCK-8 assay kit from Beyotime Biotechnology, following the manufacturer’s protocol. Cells were seeded into 96-well plates and maintained at 37 °C in a humidified 5% CO_2_ atmosphere for 24 h. Absorbance measurements were taken at 450 nm with a microplate reader.

### 2.4. Extraction and Real-Time Fluorescence Quantification of RNA

The total RNA was extracted from samples using the Trizol reagent (TaKaRa, Dalian, China), and then converted into cDNA with the PrimeScriptTM RT kit (TaKaRa) for subsequent applications. For miRNA analysis, cDNA synthesis was conducted using a stem-loop-based miRNA 1st Strand cDNA Synthesis Kit (Vazyme, Nanjing, China), followed by RT-qPCR with the miRNA Universal SYBR qPCR Master Mix (Vazyme, Nanjing, China). The quantification of fluorescence was carried out using the Light Cycler 96 instrument system (Roche, Basel, Switzerland). RT-qPCR primers were synthesized in Shanghai, China. We have utilized GAPDH and U6snRNA as endogenous reference genes, and the relative expression was calculated as the level of miRNA or mRNA using the 2^−Δ Δ CT^ method. The primers used are listed in Table 1.

### 2.5. Plasmid Construction and Cell Transfection

For the dual-luciferase assay, COL4A1 wild (COL4A1–WT) and mutant (COL4A1–MUT) strains were built according to the conjugation sites of COL4A1 and miR–122–5p, and they were cloned into a pmir–GLO vector. Then, the pmir–GLO, COL4A1–WT, and COL4A1–MUT vectors were transfected into the HEK–293T cells, respectively.

To study COL4A1 overexpression, full-length COL4A1 was amplified and digested with Hind III and Xho I (Takara, Dalian, China). T4 ligase (TaKaRa, Dalian, China) was used for the ligation of the pcDNA3.1+ vector. The plasmid overexpressing COL4A1 was extracted from a bacterial medium containing 200 μg/mL ampicillin (Beyotime Biotechnology, Shanghai, China). The miR–122–5p mimics, inhibitor, and negative control (mimics, inhibitor, and NC) were synthesized by GenePharma (Shanghai, China). For cell transfection, the NR8383 cells were seeded into 6-well plates and cultured at 37 °C in a 5% CO_2_-humidified environment. Lipofectamine 3000 (Thermo Fisher, MA, USA) was used as a transfection reagent according to the manufacturer’s instructions. The cells were collected for analysis after 48 h.

Because HEK-293T cells have higher transfection stability and efficiency than NR8383 cells do, we used Lipofectamine 3000 (Thermo Fisher, MA, USA) to simultaneously transfect the miR–122–5p mimics (or mimics NC) and COL4A1–WT (or COL4A1–MUT) in the HEK-293T cells in 6-well plates. Luciferase activity, 48 h post-transfection, was determined with a dual-luciferase reporter assay.

### 2.6. Flow Cytometry

NR8383 cells were seeded into a 6-well plate and incubated at 37 °C in a humidified 5% CO_2_ atmosphere for 24 h. After incubation, cells were washed with phosphate-buffered saline (PBS), trypsinized, and collected. Apoptosis was assessed using the Annexin V-FITC/PI kit (556547, BD PharmingenTM, Franklin Lakes, NJ, USA), and flow cytometry analysis was conducted on a BD LSRFortessa instrument (BD Biosciences, Franklin Lakes, NJ, USA).

### 2.7. Hoechst 33342/PI Double Staining

We detected apoptosis by using Hoechst 33342/PI double staining (Beyotime Biotechnology, Shanghai, China). The NR8383 cells were plated in 12–well plates, and 5 μL of Hoechst 33342 and PI staining solution was added to each well after challenge or transfection. The mixture was then evenly mixed, and fluorescence was detected at 4 °C with a fluorescence microscope (Olympus, Tokyo, Japan). The normal cells exhibited weak blue and red fluorescence under the microscope, whereas the apoptotic cells displayed weak red and strong blue fluorescence following Hoechst 33342/PI staining.

### 2.8. Western Blotting

We have prepared pre-cooled RIPA lysate, containing 1% phosphatase inhibitor and 1% protease inhibitor, to lyse cells. Protein concentration was determined using the BCA Protein Assay Kit from Beyotime Biotechnology, following the manufacturer’s protocol. The protein samples were initially separated through SDS-PAGE. Afterward, the protein was transferred onto a PVDF membrane via electrophoresis. The primary antibody was then incubated overnight at 4 °C, followed by three washes with TBST for 10 min each time and the secondary antibody at a concentration of 1:8000 was then added to the sample. Finally, an ECL luminescent solution (Beyotime Biotechnology, Shanghai, China) was used to develop the color. The captured image was analyzed using Image J 1.8.0 software (USA).

The antibodies used in this study, all at a dilution of 1:1000 unless stated otherwise, were sourced from Proteintech (Philadelphia, PA, USA) for BCL-2, BAD, AKT, p-AKT, IκBα, and GAPDH. PI3K was used at 1:5000, while β-actin was at 1:900. Antibodies sourced from Abways (Shanghai, China) include p-PI3K (1:1000) and p-IκBα (1:500), along with p-P65 (1:500) and p-P65 (1:500). Caspase-3 was obtained from Santa cruz Biotechnology (Santa Cruz, CA, USA) at a dilution of 1:1000, and COL4A1 was obtained from Thermo Fisher Scientific (Waltham, MA, USA), also at 1:1000. Secondary antibodies, anti-rabbit IgG (1:8000), and horseradish peroxidase-labeled goat anti-mouse IgG (1:8000), were also from Proteintech.

### 2.9. Statistical Analysis

Study data were analyzed using GraphPad Prism 8.0 (La Jolla, CA, USA), with *t*-tests for two–group comparisons and one–way ANOVA for multiple groups. A *p*-value of <0.05 indicated statistical significance, confirmed by triplicate experiments.

## 3. Results

### 3.1. Cowshed PM_2.5_ Exposure Induces Apoptosis in NR8383 Cells

**Figure 1:**
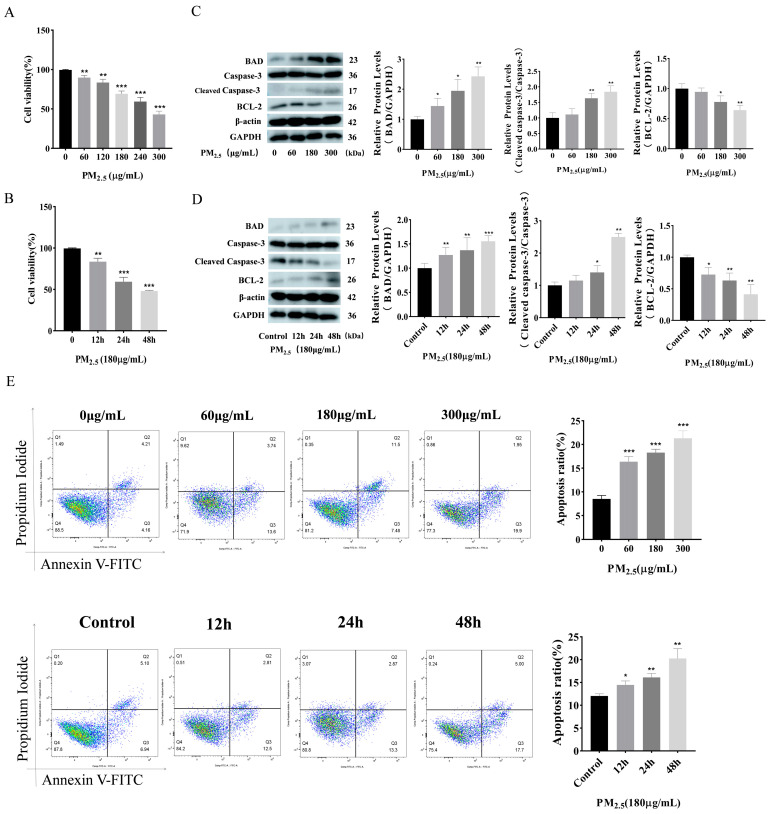
Apoptosis induced by cowshed PM_2.5_ exposure was assessed. (**A**,**B**) A CCK–8 assay was used to evaluate cell viability under varying concentrations and durations of exposure. (**C**,**D**) The alterations in apoptosis-associated proteins, including BAD and levels of Caspase–3 and BCL–2, were evaluated using Western blotting across various concentrations and time intervals. (**E**) The detection of apoptosis using flow cytometry. Asterisks denote the levels of significance as detailed below: * *p* < 0.05; ** *p* < 0.01; and *** *p* < 0.001.

Initially, our research group analyzed PM_2.5_ components and community components in cowsheds [27,28]. The cell viability decreased with an increasing concentration of cowshed PM_2.5_ compared to the control group, as demonstrated by the use of the cell Counting Kit–8 (CCK–8) results (Figure 1A,B). The cell viability was decreased to less than 50% at the exposure concentration of 300 μg/mL. Therefore, we chose 180 μg/mL for the subsequent experiments. Figure 1C shows that the expression levels of apoptotic BAD and Caspase–3 proteins, and that of the anti-apoptotic BCL–2 proteins increased significantly in the NR8383 cells stimulated with different concentrations of PM_2.5_. Figure 1D shows that the changes in apoptosis protein levels in the NR8383 cells stimulated by cowshed PM_2.5_ at different times are consistent with those of the proteins mentioned above. Using flow cytometry (Figure 1E) and the Hoechst 33342/PI staining assay (Figure S1), we also reached the same conclusion. The above experimental results show that cowshed PM_2.5_ can reduce cell viability and promote apoptosis.

### 3.2. Cowshed PM_2.5_-Stimulated Upregulation of miR–122–5p

**Figure 2:**
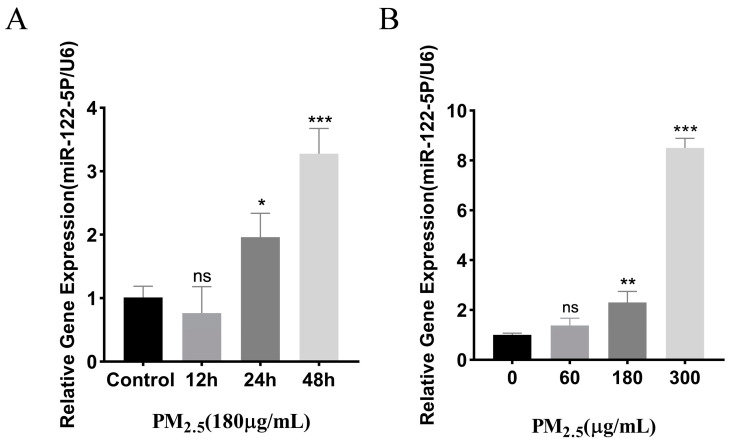
The expression of miR–122–5p stimulated by cowshed PM_2.5_. (**A**,**B**) The detection of miR–122–5p using an RT-qPCR stimulated by cowshed PM_2.5_ at different time periods (0 h, 12 h, 24 h, and 48 h) and across a range of concentrations from 0 to 300 μg/mL. Asterisks mark statistical significance: ns *p* > 0.05; * *p* < 0.05; ** *p* < 0.01; and *** *p* < 0.001.

Our group established a rat model in the early stage [29]. Through screening, we found that there were differential expressions of the microRNAs (miRNAs). There were significant differences in the expression of miR–122–5p (Table S1). We investigated this based on its biological characteristics. miR–122–5p levels in NR8383 cells, following stimulation with cowshed PM_2.5_ across varying concentrations and time points, were confirmed through RT-qPCR analysis. (Figure 2A,B). The results show that the miR–122–5p expression level was increased when different concentrations of cowshed PM_2.5_ were applied. In addition, the expression of miR–122–5p was upregulated in cells stimulated at the same concentration over different time periods. These findings suggest a link between miR–122–5p expression and fluctuations in PM_2.5_ exposure, underscoring the necessity for investigating the underlying molecular mechanisms.

### 3.3. Promotion of Apoptosis by miR–122–5p

**Figure 3:**
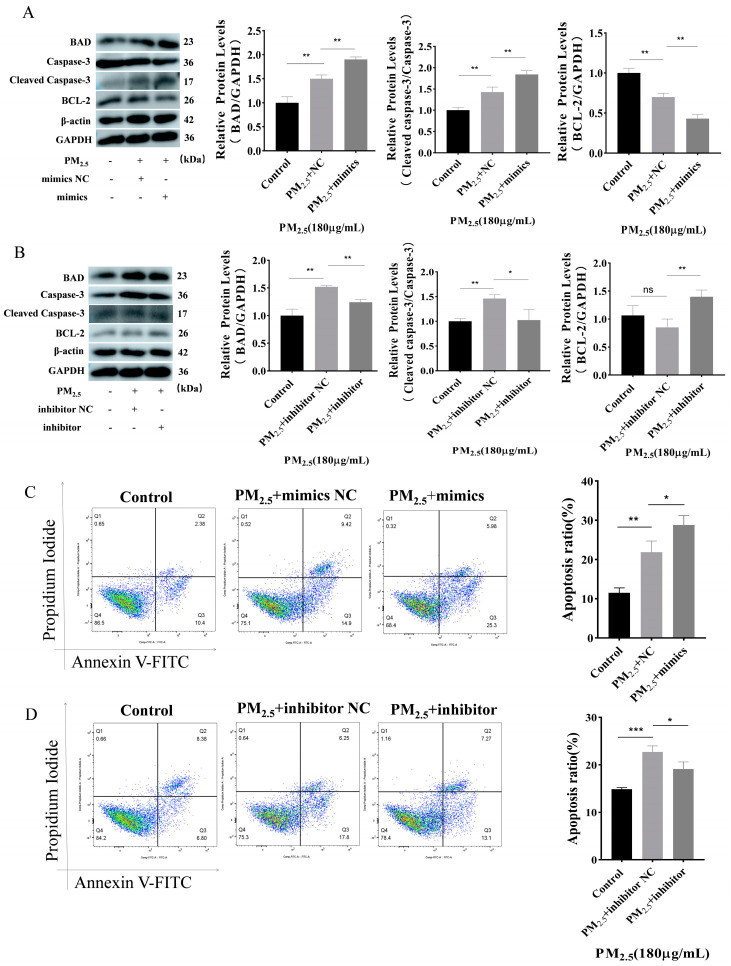
Apoptosis promoted by miR–122–5p. (**A**) Effects of NC and miR–122–5p mimics transfection on apoptosis proteins detected under PM_2.5_ challenge. (**B**) Changes in apoptotic proteins after transfection with inhibitor NC, and miR–122–5p inhibitors examined under PM_2.5_ exposure. (**C**) Apoptosis induced by NC and miR–122–5p mimics was assessed through flow cytometry. (**D**) Impact of inhibitor NC and miR–122–5p inhibitor on apoptosis was determined through flow cytometry. Asterisks denote levels of significance as detailed below: ns *p* > 0.05; * *p* < 0.05; ** *p* < 0.01; and *** *p* < 0.001.

This study found that miR–122–5p mimics upregulated the expression of apoptosis-promoting proteins while downregulating the expression of the anti-apoptotic BCL–2 proteins (Figure 3A). Conversely, miR–122–5p inhibitor had a contrary result compared to miR–122–5p mimics (Figure 3B). The flow cytometry results also support the aforementioned findings (Figure 3C,D). Similarly, Hoechst 33342/PI staining has reflected the same trend (Figure S2). In the presence of cowshed PM_2.5_, the presence of miR–122–5p mimics has led to an increase in apoptosis, as indicated by these results. Inversely, the incorporation of a miR–122–5p inhibitor has been shown to reverse apoptosis. This suggests that miR–122–5p has the potential to promote apoptosis.

### 3.4. COL4A1 Is a Predicted Target of miR–122–5p and Downregulated in Cowshed PM_2.5_-Induced NR8383 Cells

**Figure 4:**
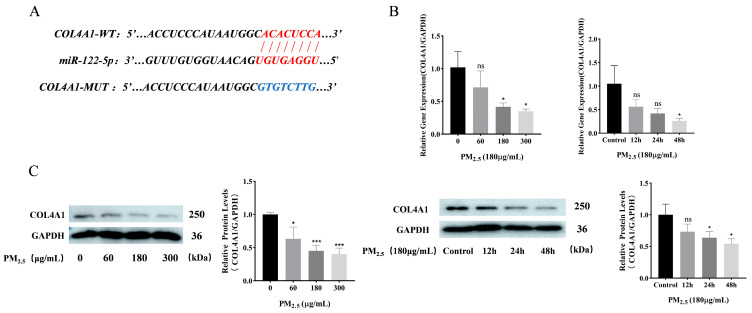
COL4A1, a putative target of miR–122–5p, exhibits downregulated expression when exposed to PM2.5 from cowshed environments. (**A**) The target has predicted the combined position between miR–122–5p and *COL4A1*. (**B**) Changes in COL4A1 were identified following the PM_2.5_ treatment, as determined by RT-qPCR. (**C**) The changes in *COL4A1* were detected using Western blotting under different concentrations and different time periods of PM_2.5_ exposure. Significance is indicated by asterisks, as detailed below: ns *p* > 0.05; * *p* < 0.05; and *** *p* < 0.001.

Using miRDB microRNA target prediction database and Targetscan 7.2 (http://www.targetscan.org/vert_80/) (accessed on 1 February 2023), the fraction that binds to miR–122–5p was identified in the 3′UTR region for *COL4A1* (Figure 4A). Molecular and protein expression analyses were performed to evaluate COL4A1 levels post-PM_2.5_ stimulation. These results indicated that the *COL4A1* expression level was reduced in a concentration- and time-dependent manner (Figure 4B,C).

### 3.5. miR–122–5p Targeted COL4A1 and Showed Negative Regulatory Relationship

**Figure 5:**
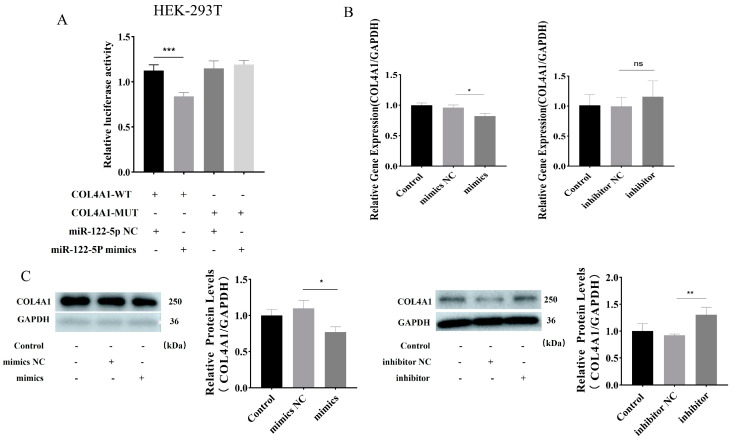
Negative regulation of miR–122–5p and *COL4A1*. (**A**) Binding of miR–122–5p to COL4A1 was verified by dual-luciferase experiments using 293T cells. (**B**) Analysis of *COL4A1* expression after transfer of mimics NC, miR–122–5p mimics, inhibitor NC, and miR–122–5p inhibitor using RT–qPCR. (**C**) Western blot detection of *COL4A1* protein expression after transfection of NC, miR–122–5p mimics, inhibitor NC, and miR–122–5p inhibitor. Asterisks denote levels of significance as detailed below: ns *p* > 0.05; * *p* < 0.05; ** *p* < 0.01; and *** *p* < 0.001.

The dual-luciferase assay results indicate that miR–122–5p mimics diminish luciferase activity relative to controls, suggesting that miR–122–5p targets COL4A1 (Figure 5A). The regulatory link between miR–122–5p and COL4A1 was confirmed, with results showing COL4A1 downregulation upon miR–122–5p mimic introduction (Figure 5B). Moreover, COL4A1 expression was elevated by the miR–122–5p inhibitor, as demonstrated by the Western blot and RT-qPCR analyses (Figure 5C). In summary, miR–122–5p binds to COL4A1, establishing a negative regulatory relationship, as evidenced by the data presented.

### 3.6. Overexpression of COL4A1 Enhances PI3K/AKT/NF–κΒ Signaling Pathway and Inhibits Cowshed PM_2.5_-Induced Apoptosis

**Figure 6:**
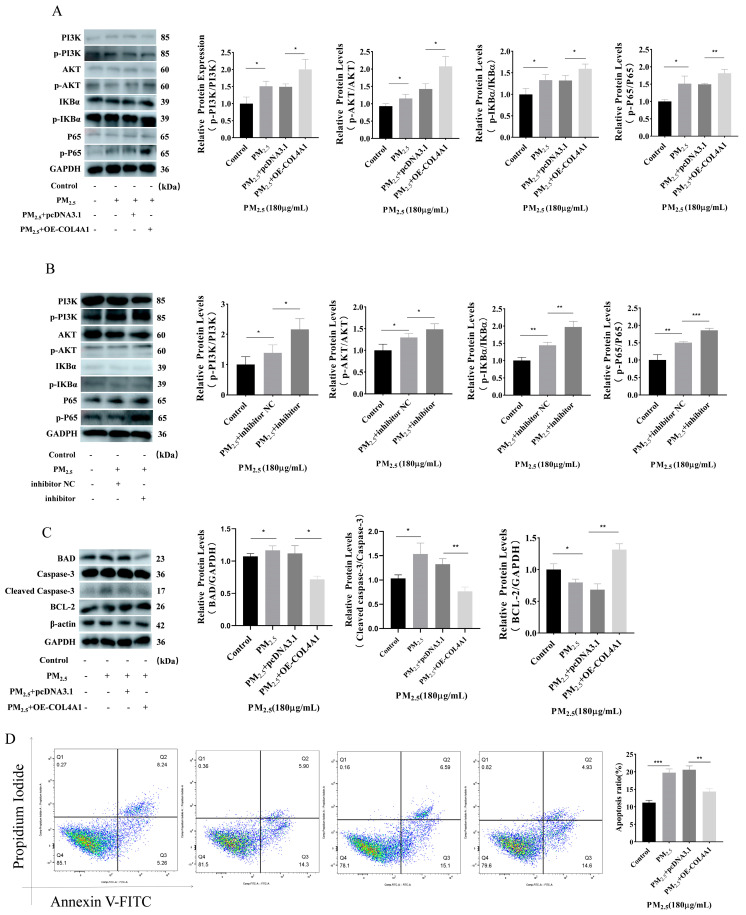
Overexpression of *COL4A1* activates the PI3K/AKT/NF–κΒ pathway and inhibits apoptosis induced by cowshed PM_2.5_. (**A**) Effect of OE–*COL4A1* on PI3K/AKT/NF–κΒ pathway proteins under cowshed PM_2.5_ exposure. (**B**) Impact of miR–122–5p inhibitor against PI3K/AKT/NF–κΒ pathway under cowshed PM_2.5_ exposure using Western blotting. (**C**) Effect of *COL4A1* overexpression on apoptotic proteins under cowshed PM_2.5_ exposure as determined using Western blot. (**D**) Apoptosis levels in COL4A1–overexpressing cells were evaluated using flow cytometry. Asterisks denote significance levels: * *p* < 0.05; ** *p* < 0.01; and *** *p* < 0.001.

The overexpression of *COL4A1* can enhance the PI3K/AKT pathway activated by cowshed PM_2.5_. At the same time, the quantity of phosphorylated IKBα increased, and phosphorylated P65 was activated, thereby activating the NF–κΒ pathway (Figure 6A). Subsequently, we investigated the potential regulatory role of miR–122–5p within the signaling pathway induced by cowshed PM_2.5_. Because the negative regulatory relationship between miR–122–5p and *COL4A1* was determined, we used a miR–122–5p inhibitor to verify it. The results show that the miR–122–5p inhibitor had an effect on the pathway under the action of cowshed PM_2.5_ (Figure 6B). Then, we detected changes in the apoptosis proteins in this pathway. The results show that with the increase in the *COL4A1* expression level, the expression of the apoptosis proteins BAD and Caspase–3 were downregulated, and the expression of BCL–2 was upregulated (Figure 6C). The flow cytometry trend was also consistent with these findings (Figure 6D). We also verified this using Hoechst 33342/PI (Figure S3). These results indicated that the overexpression of *COL4A1* can regulate apoptosis by activating the PI3K/AKT/NF–κΒ pathway.

## 4. Discussion

In this study, cells treated with cowshed PM_2.5_ showed reduced viability with amplified apoptosis. The changes in cell viability and apoptosis occurred in a concentration- and time-dependent manner and are consistent with Ming’s findings [30]. The level of PM_2.5_ in livestock and poultry houses impacts economic development and indoor breeding benefits. Hence, a comprehensive understanding of PM_2.5_ pathogenesis in animal breeding environments is crucial. Consequently, this study has elucidated the apoptotic mechanism triggered by PM_2.5_ exposure.

Exposure to PM_2.5_ can lead to alterations in the microRNA [31]. miR–122–5p is associated with numerous human diseases, such as cancer and obesity. It can regulate the cell epithelial–mesenchymal transition and oxidative damage [32,33,34]. Changes in miR–122–5p due to cowshed PM_2.5_ are infrequently reported. Thus, a potential link between miR–122–5p and cowshed PM_2.5_ exposure was hypothesized. In this research, we discovered that the alterations in miR–122–5p under cowshed PM_2.5_ exposure are both concentration- and time-dependent. Our findings show that miR–122–5p mimics enhance PM2.5-induced cell death, while the miR–122–5p inhibitor blocks this effect, confirming the findings in earlier studies [35]. Our speculation suggests a possible association between miR–122–5p and apoptosis, which was the motivation for our investigation. To investigate the promotion of apoptosis by miR–122–5p, we have predicted the target gene and discovered its possible correlation with *COL4A1*. Subsequently, we have confirmed that a targeted negative regulatory relationship exists between *COL4A1* and miR–122–5p. miRNA targets the 3′ UTR of *COL4A1* and regulates apoptosis by controlling its transcription and translation. Additionally, it was reported that miR–124 inhibits the epithelial–mesenchymal transition in gastric cancer cells via its target *COL4A1* [36]. Megan Griffiths and colleagues have discovered that the upregulation of miR–29c is associated with a reduction in *COL4A1* expression [37]. These studies affect cell function by binding the microRNA and messenger RNA. Nevertheless, the impact of miR–122–5p-targeted *COL4A1* on cell function requires further investigation.

A considerable amount of evidence has shown that there are many types of type I collagen, and they are associated with respiratory diseases [38]. Other studies indicate significant differences in the collagen type IV expression levels between people with pulmonary fibrosis and chronic obstructive pulmonary disease (COPD) [39]. However, certain studies have found a decreased expression of collagen type IV in people with pulmonary fibrosis [40]. Our findings demonstrate that cowshed PM_2.5_ reduces the expression level of *COL4A1*. It is hypothesized that cowshed PM_2.5_ may impact the changes in *COL4A1* due to the reduced content of collagen type IV in cells resulting from external stimuli. Furthermore, varying expression levels across different cells may be related to the changes observed in *COL4A1*. Prior to assembly, collagen morphology is synthesized within the cells and is subsequently involved in the extracellular matrix formation. Collagen type IV, as the primary component of the ECM, interacts with the internal and external cell environments through integrin [41]. The diminished expression of collagen type IV can result in extracellular matrix degradation, which destabilizes the matrix structure. The overabundance or degradation of the ECM will impact the cell structure; hence, we can infer that the condition of the cells may be affected in this manner [42]. As a member of the ECM family, the relationship between COL4A1 and the respiratory system changes in real time. For different diseases, COL4A1 has a dynamic impact on them. The research to date indicates a correlation between the production of type IV collagen and the severity of pulmonary fibrosis. When there is an increase in collagen, this is a sign of fibrosis in a sense. At the same time, collagen can also cause other proteins to affect other diseases; for example, airway inflammation will be inhibited as collagen changes the MUC5AC protein [14]. MiR–122–5p and *COL4A1* exhibited a targeted negative regulatory relationship, indicating that *COL4A1* regulation is influenced by multiple factors. These findings offer a novel perspective on the miRNA targeting of collagen family-mediated apoptosis.

Based on our research, we have identified a miRNA that negatively regulates the targeting of *COL4A1*. We then investigated the pathways that impact apoptosis and the potential mechanism of *COL4A1* in this process. Using bioinformatics analysis, we have specifically located the PI3K/AKT/ NF–κΒ pathway. Excessive and suppressed PI3K/AKT signaling has been linked to various pathologies. [43,44]. The activation of the PI3K/AKT pathway has been reported to induce the epithelial–mesenchymal transition and the proliferation of epithelial cells [45]. Another study has discovered that the peptide *COL4A1* mitigates the development of eclampsia by affecting the TGF-β/PI3K/AKT pathway [46]. The PI3K/AKT pathway is a well-known apoptosis route that is closely associated with apoptosis [47]. However, the effect of the NF-κΒ signaling pathway is complicated [48]. In our study, it was discovered that the augmentation of *COL4A1* expression initially triggers the PI3K/AKT pathway, which subsequently activates the NF–κΒ pathway, resulting in the inhibition of apoptosis. This may result from an NF–κB response element (KB site) on the BCL–2 protein upon initiation of the NF-κB signaling pathway. This led to the upregulation of the anti-apoptotic protein expression in BCL–2. Likewise, the impact of NF-κΒ on apoptosis varied across the different cells. The Caspase family plays a crucial role in apoptosis, with Caspase-3 serving as the primary executor. Our Western blot analysis has revealed the downregulation in Caspase–3 gene expression, confirming our hypothesis that *COL4A1* overexpression hinders cowshed PM_2.5_-induced epithelial cell death with the activation of the phosphatidylinositol–3–kinase (PI3K) and protein kinase B (AKT) signaling cascade, culminating in the upregulation of the nuclear factor kappa B (NF–κB) pathway. Furthermore, the miR–122–5p inhibitor can both regulate COL4A1 expression and activate the aforementioned pathways through this modulation.

## 5. Conclusions

In summary, our research indicates that cowshed PM_2.5_ can trigger NR8383 apoptosis. Additionally, our research indicates that miR–122–5p triggers the PI3K/AKT signaling cascade, which in turn modulates the NF–κB pathway by binding to COL4A1, thereby suppressing apoptosis. This discovery presents a fresh perspective and a novel therapeutic target for PM_2.5_-induced lung injuries.

## Figures and Tables

**Table 1:** The primers for RT-qPCR.

Gene	Primers’ Sequence (5′-3′)
*COL4A1*	F: AGTTGGCTTTCCTGGTAGTC R: AAGGCCTGCTTGTCCTTT
*GAPDH*	F: CCTGCACCACCAACTGCTTA R: CATCACGCCACAGCTTCCA
U6	F: CTCGCTTCGGCAGCACA R: AACGCTTCACGAATTTGCGT
miR–122–5p	F: CGCGTGGAGTGTGACAATGG R: AGTGCAGGGTCCGAGGTATT

## Data Availability

The data presented in this study are available on request from the corresponding authors.

## Supplementary Materials

The following supporting information can be downloaded at: https://www.mdpi.com/article/10.3390/toxics12060386/s1. Figure S1: Hoechst33342/PI was used to detect cell apoptosis at different times and different concentrations; Figure S2: Hoechst33342/PI was used to detect the effect of miR–122–5p on cell apoptosis; Figure S3: Hoechst33342/PI was used to detect the effect of OE-COL4A1 on cell apoptosis; Table S1: Differentially expressed miRNAs.
