# Supplementary material for: miR–122–5p Promotes Cowshed Particulate Matter2.5-Induced Apoptosis in NR8383 by Targeting COL4A1
Source: Toxics. 2024 May 25;12(6):386. doi: 10.3390/toxics12060386 (PMC11209608; doi:10.3390/toxics12060386)
Supplement: Supplementary file 1 [file toxics-12-00386-s001.zip › Figure S1.pdf]

S1

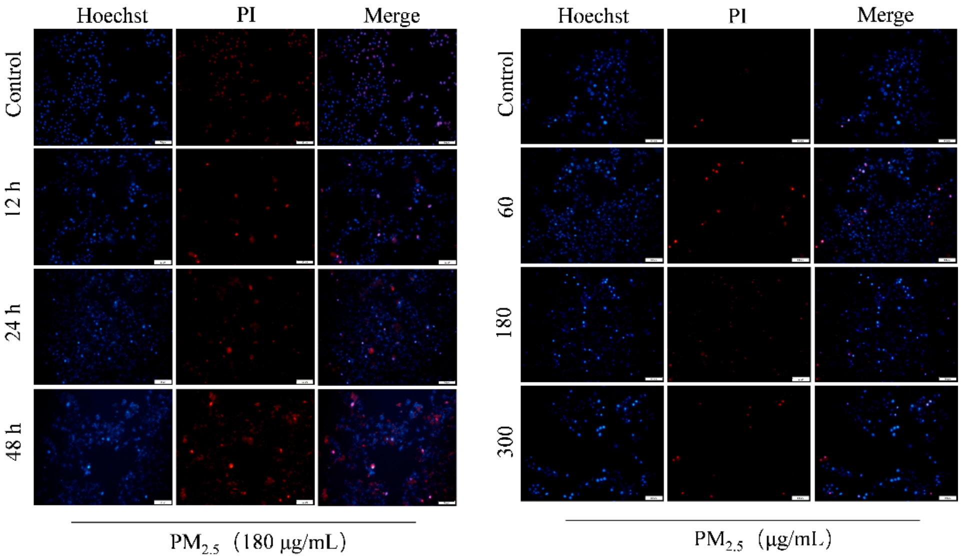

Figure S1. Hoechst33342/PI was used to detect cell apoptosis at different times and different concentrations.
