# Supplementary figures and images for: miR–122–5p Promotes Cowshed Particulate Matter2.5-Induced Apoptosis in NR8383 by Targeting COL4A1
Source: Toxics. 2024 May 25;12(6):386. doi: 10.3390/toxics12060386 (PMC11209608; doi:10.3390/toxics12060386)

S2

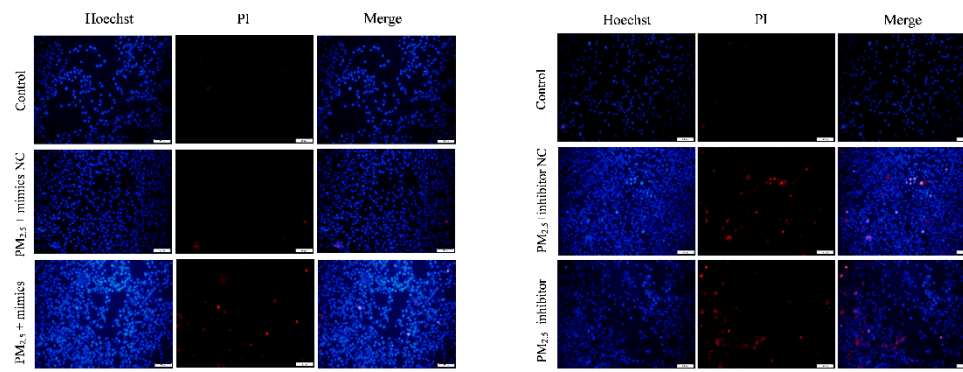

Figure S2. Hoechst33342/PI was used to detect the effect of miR-122-5p on cell apoptosis.

Supplement: Supplementary file 1 [file toxics-12-00386-s001.zip › Figure S2.pdf]

S3

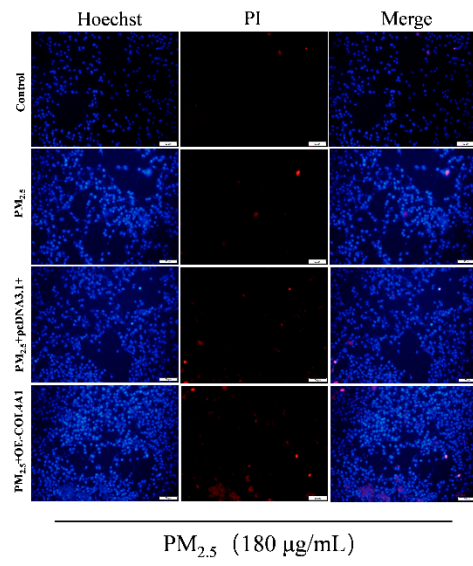

Figure S3. Hoechst33342/PI was used to detect the effect of OE-COL4A1 on cell apoptosis.

Supplement: Supplementary file 1 [file toxics-12-00386-s001.zip › Figure S3.pdf]
